# Social support and social comparison tendencies predict trajectories of adolescents’ problematic social media use: A longitudinal study

**DOI:** 10.1371/journal.pone.0323320

**Published:** 2025-06-18

**Authors:** Laura Salerno, Lucia Fortunato, Vivian Ostwald, Arianna Muscolino, Gianluca Lo Coco

**Affiliations:** 1 Department of Psychology, Educational Sciences and Human Movement, University of Palermo, Italy; 2 Department of Clinical Psychology and Psychotherapy, Ruhr-University Bochum, Bochum, Germany; Tianjin University, CHINA

## Abstract

There is still no scientific consensus on the relationship between problematic social media use and its negative consequences for adolescents’ well-being. This study aims to identify common trajectories of problematic social media use over time in a sample of adolescents, and to identify the demographic and psychological factors associated with different empirical profiles. This four-wave longitudinal study involved 403 adolescents (age range: 13–18 years; M_age_ = 15.73 ± 1.22; 51.9% females). Participants provided demographic and social media use data (i.e., social media addiction, frequency of social media activities, time spent on Instagram and TikTok) as well as measures of psychological factors (i.e., psychological distress, emotion dysregulation, self-esteem, perceived social support, online social comparison). Parallel latent class growth analysis (LCGA) categorised participants into three classes. Participants in class 1 (‘most vulnerable group’; 25.56%) showed a more impaired functioning profile, with a stable problematic pattern of social media use (i.e., high levels of social media addiction and time spent on TikTok). Multinomial regression models showed that factors associated with this pattern were low social support, high online social comparison, and SMU intensity. Our findings suggest that the role of social interactions (both online and offline) and social comparison tendencies should be further explored as markers of problematic social media use.

## Introduction

In recent years, increasing concerns have been raised about the potential negative impact of social media use on the well-being of adolescents [1,2]. The vast majority of youth report daily use of social media through their mobile devices, and a large percentage of them report being online almost constantly [3,4]. Although social media such as Instagram, Snapchat or TikTok allow adolescents to build and maintain social relationships through online features such as sharing personal photos and videos or through instant messaging, there is a call for research investigating problematic social media use and its negative consequences for individual well-being among young people [5,6]. Some recent reviews highlighted a small negative association between social media use and depression, poor well-being and loneliness among young adults [7,8]. Specifically, it has been argued that time spent on social media, frequency of checking social media, or intensity of use may represent markers of social media addiction, with users experiencing a lack of regulation and various impairments in life functioning [9,10]. However, the construct of social media addiction among adolescents remains problematic and has not been recognised as such in any diagnostic manual [10,11,12]. Although some evidence suggests that excessive social media use may lead to symptoms traditionally associated with addiction [9], previous research on the interaction between social media addiction and time spent online among adolescents has reported inconsistent findings [13]. A recent review of 233 articles focusing on the impact of social media use on well-being found that 72% of studies examined the impact of social media use by assessing the time users spent on social media, while 28% examined the impact of problematic social media use or addiction on well-being [14].

Although most concerns about problematic social media use have focused on screen time, there is still no scientific consensus on the impact of screen-based activities on young people’s mental health and well-being [15,16]. For example, a study of a large representative sample of 14-year-olds living in the UK found an association between social media use and depressive symptoms, and that this association was stronger for girls than for boys [17]. In addition, an international survey found that problematic social media use was associated with lower mental and social well-being among adolescents in 29 countries [18]. However, other studies found limited evidence for a negative causal relationship between digital screen engagement and adolescent well-being [19,20]. For example, Orben & Przybylski [20] examined three nationally representative large-scale datasets, including time-use diary measures of digital screen engagement, and found little evidence of a negative association between digital screen engagement and adolescent well-being. Overall, the most recent reviews of studies examining the association between screen time and mental health symptoms in young people highlighted a small to very small association between screen time and depression, and a weak association between screen time and both anxiety and self-esteem [5,21]. The review by Tang et al. [21] examined longitudinal studies and suggested that the relationship between screen time and mental health is complex and varies across different types of screen devices and uses. For example, previous research has tended to focus on social media use in general. However, the Multidimensional Model of Social Media Use [22] suggests that: a) individual platforms differ in several ways; b) social media use varies by activity (e.g., active posting, passive browsing), motives for use (e.g., enhancement or compensation), and communication partners (e.g., strong or weak ties). In addition, some concerns have been raised about the validity of self-reported data on digital media use [23,24], given evidence that young people tend to under- or overestimate their use of social media, and there are calls for research that can employ more nuanced methodologies other than self-report assessments [25,26].

Despite these differences in social media platform activities and interaction partners, few studies have examined how adolescent characteristics may be more relevant to some platforms than others [27]. For example, it was shown that less extraverted adolescents and those with a tendency to react negatively to social media reported increased depressive symptoms when they used Instagram and TikTok more frequently [28]. Investigating how social media use is associated with negative mental health outcomes for some adolescents but not others is recognised as an important research task [29]. It is also worth noting that limited research has explored how technology addiction or problematic social media use develops over time [30,31].

A recent study [32] examining the trajectories of adolescents’ problematic social media use and social media use frequency showed that different subgroups could be identified and that levels of problematic social media use remained high over a four-year period for those groups with higher engagement with social media. However, in the largest subgroup, adolescents reported persistently low levels of problematic social media use with variably high levels of social media use frequency. Thus, further research is needed to examine how excessive social media use is intertwined with problematic social media use over time [18]. Problematic social media use among adolescents is associated with relational, emotional, and performance problems, which may influence an individual’s motivation to overuse social media in the short term [9]. Analysing short-term longitudinal changes in problematic social media use is therefore a promising area of research, and there is some preliminary evidence that problematic internet use can change over the course of a few weeks (see 34).

It is also worth noting that individual differences should be taken into account when examining developmental patterns of social media use among adolescents. Adolescence is characterized by a complex process of identity formation and the development of social skills and internal management of emotions play an important role in social adjustment [33,34]. A positive and stable evaluation of the self is also an important developmental task that adolescents need to achieve through peer interactions and feedback to the self. Young people’s engagement with social media is embedded in these developmental tasks. Adolescents typically spend several hours in social media to interact with their peers by receiving feedback on the self [35]. Some theoretical models suggest that lonely adolescents with poor social support may turn to social media engagement to compensate for their lack of interpersonal relationships and to manage their negative emotions [36,37]. Moreover, adolescents who spend more time on social media are more likely to report lower level of self-esteem [38], by experiencing negative feedback on the self. Prior research suggests that social comparison orientation [39] and emotion regulation strategies [22] may be associated with dysfunctional patterns of social media use. Although adolescents tend to maintain social relationships through social media use, their widespread comparison of appearance (i.e., how my body looks compared to other teens) on appearance-focused social media such as Instagram or TikTok may be associated with problematic use [40,41]. It has also been argued that problematic social media use may be associated with reduced real-life social support [42,43]. Adolescents may tend to engage in social media to withdraw from the ‘social pain’ of interaction, and feelings of social isolation are likely to occur [44,45]. Finally, problematic social media users are more likely to report higher levels of emotional dysregulation [46,47] and it was suggested that adolescents with difficulties in emotion regulation may be more likely to become involved in problematic internet behaviors and social media use as an attempt to manage negative emotions.

The aim of the present study is to extend the literature on the negative consequences of social media use among adolescents. Specifically, the study has two aims: a) to identify joint longitudinal trajectories (or co-developments) of problematic social media use (i.e., social media addiction) and objective data on daily time spent on Instagram and TikTok, by analysing four waves of panel data from a sample of Italian adolescents; b) to identify the demographic and psychological characteristics associated with the different empirical profiles. Given the importance of developing emotional and social competencies during adolescence, we examined whether emotion regulation, social support, self-esteem and social comparison might be associated with different trajectories of problematic social media use.

## Materials and methods

### Participants and procedures

The current study is part of a larger project on the impact of social media use during the COVID-19 pandemic. For the purposes of this study, four-wave panel data were collected from 403 adolescents (M_age_ = 15.73 ± 1.22; 51.9% female) (see Table 1 for participants’ sociodemographic data at the beginning of the study – T0). The target group of this study included adolescents aged between 13 and 18 years, who owned a smartphone and had sufficient knowledge of the Italian language to understand the study questionnaires. Participants were recruited from two high schools in Italy (i.e., Palermo and Naples) and selected on the basis of their availability using convenience sampling. Data collection took place in September 2022 (T0), October 2022 (T1), November 2022 (T2) and April 2023 (T3). The longer time interval between T2 and T3, compared to the interval between the first three measurements, is due to the specific needs of the schools involved in the study. However, as all participants entered the study at the same time, and the data were available at exactly the same time, even if the measurement times were at different intervals, no special control was necessary within the procedure used. Written parental consent was obtained at T0. Participation in the study was voluntary and unpaid, and participants were informed that they could withdraw at any time. The study was conducted according to the guidelines of the Declaration of Helsinki and approved by the Ethics Committee of the University of Palermo Ethics Committee (Nr. 86/2022–26 May 2022).

**Table 1:** Participants’ (n = 403) sociodemographic data at T0.

	M(SD)/n(%)
Age, M (SD)	15.73 (1.22)
Gender, n (%)	
* Males*	122 (30.2%)
* Females*	209 (51.9%)
* missing*	72 (17.9%)
Engagement status, n (%)	
* single*	241 (59.8)
* in a relationship*	82 (20.3)
* missing*	80 (19.9)
City of residence, n (%)	
* *Naples	208 (51.6)
* *Palermo	195 (48.4)
School class, n (%)	
* *High school, I year	76 (18.9)
* *High school, II year	90 (22.3)
* *High school, III year	130 (32.2)
* *High school, IV year	107 (26.6)

### Measures

#### Baseline assessment.

Participants’ socio-demographic characteristics (i.e., age, gender, engagement status, city of residence and school class) were collected in the first part of the questionnaire. Psychological distress was measured using the Young Person’s CORE (Clinical Outcomes in Routine Evaluation; YP-CORE) [48]. The YP-CORE consists of 10 items (e.g., My thoughts and feelings bothered me) that are rated on a 5-point Likert scale (ranging from 0 = not at all to 4 = most or all of the time). In the present study, the YP-CORE showed good reliability (Cronbach’s alpha = .809). Emotional dysregulation was measured using the Difficulties in Emotion Regulation Scale (DERS-18) [49]. The DERS consists of 18 items (e.g., When I’m upset, I think I’ll stay that way for a long time) that are rated on a five-point Likert scale (ranging from 1 = almost never to 5 = almost always). In the present study, only the DERS-18 total score was used, which showed good internal reliability (Cronbach’s alpha = .860). Self-esteem was measured using the Rosenberg Self-Esteem Scale (RSES) [50]. The RSES consists of 10 items (e.g., I feel that I have a number of good qualities) that are rated on a 4-point Likert scale (ranging from 1 = strongly agree to 4 = strongly disagree). In the present study, the RSES showed good internal consistency (Cronbach’s alpha = .875). Perceived social support was measured using the Multidimensional Scale of Perceived Social Support (MSPSS) [51]. The MSPSS consisted of 12 items (e.g., There is a special person in my life who cares about my feelings) that are rated on a 7-point Likert scale (ranging from 1 = strongly disagree to 7 = strongly agree). In the present study, only the MSPSS total score was used, which showed excellent internal reliability (Cronbach’s alpha = .929). Online social comparison was measured using the Iowa-Netherlands Comparison Orientation Measure (INCOM) [52]. The INCOM consists of 11 items (e.g., I often compare how I am doing socially (e.g., social skills, popularity) with other people) that are rated on a 5-point Likert scale (ranging from 1 = I strongly disagree to 5 = I strongly agree). In the present study, only the INCOM total score was used, which showed good internal consistency (Cronbach’s alpha = .769). The frequency of respondents’ social media activities (SMUint) was measured by four items (i.e., “How many times a day do you visit social network sites”, “How many times a week do you ‘like’ messages, photos or videos of others on social network sites”, “How many times a week do you reply to messages, photos or videos of others on social network sites”, and “How many times a day do you send a message, photo or video via your smartphone, e.g., WhatsApp, chat, Snapchat or SMS”) [18,53]. The items were rated on a 7-point Likert scale (ranging from 1 = never or less than once and less than once for the first three items and the fourth item, respectively, to 7 = more than 40 times and more than 80 times for the first three items and the fourth item, respectively). In the present study, the scale showed good internal consistency (Cronbach’s alpha = .809).

#### Repeated assessments.

Time spent on Instagram and TikTok during the last week preceding the survey (hours per day; daily average) was objectively measured by asking participants to report data provided by their IOS or ANDROID devices. Specifically, participants were asked to report only the number of hours they received from their devices on a Likert scale from 1 (*1 hour*) to 10 (*10 hours or more*). Data on time spent on each platform is collected through a system called ‘app activity monitoring,’ which records the total time spent on the device. This system tracks time spent actively using the app, as well as scrolling and idle time, but only when the app is open and displayed on the main screen. Notably, it does not take into account apps running in the background (e.g., receiving messages or tracking location), as they do not affect the recorded usage time. When a user accesses an application via a browser on the monitored device, the system prompts the user to switch to the application, ensuring that usage time is correctly recorded. To avoid recording usage time, the user must access the application from a different device. In addition, the Bergen Social Media Addiction Scale (BSMAS) [54,55] was used to measure participants’ level of addictive use of social media. The BSMAS consists of 6 items (e.g., In the last week, have you used social media so much that it has had a negative impact on your work/studies?) that are rated on a 5-point Likert scale (ranging from 1 = very rarely to 5 = very often). Possible BSMAS scores range from 6–30, with higher scores indicating higher problematic social media use. In the present study, the BSMAS showed good internal consistency (Cronbach’s alpha: T0 = .755, T1 = .823, T2 = .825, T3 = .777).

### Data analysis plan

Normality of continuous variables was tested using skewness and kurtosis. All continuous variables had a normal distribution (|Sk| < 1 and |Ku| < 1) [56]. The internal consistency of the scales was calculated using Cronbach’s alpha. Descriptive statistics (i.e., mean and standard deviation for continuous variables and frequency and percentage for categorical variables) were calculated for demographics and variables of interest. Data were also checked for missing values. Little’s MCAR test indicated that the missing values were not missing at random (χ^2^ = 139.691, p < .001). No significant differences were found in demographics (i.e., age, gender, and engagement status), nor in psychological distress, emotion dysregulation, self-esteem, perceived social support, online social comparison, and SMU intensity at T0, nor in levels of addictive use of social media at T1 and T3 between participants with complete data in at least three waves and those with missing data in two or more waves. Significant differences were found only for participants’ school class (p < .001) and level of addictive use of social media at T0 (p < .05) and T2 (p < .05). Missing data were handled using full information maximum likelihood (FIML), which has been shown to perform better than data deletion-based methods in reducing bias in longitudinal studies, even with high rates of missing data [57].

For the first aim of the study, joint trajectories (or co-development) were generated using a three-process parallel latent class growth analysis (LCGA) [58].

Parallel process LCGA is a data-driven technique that extends typical univariate LCGA to a parallel process approach, considering multiple growth trajectories simultaneously through a small number of classes. Trajectory classes are operationalized as groups of individuals who follow approximately the same developmental trajectory. Akaike Information Criteria (AIC), Bayesian Information Criteria (BIC), sample size adjusted BIC (ssaBIC), the size of the smallest class (>5%) and entropy were used to decide the optimal number of classes. Theoretical coherence was also considered.

For the second aim of the study, multinomial regression models were run, while adjusted odds ratios (ORs) and 95% confidence intervals (CIs) were calculated to examine risk factors [baseline sociodemographic characteristics (i.e., age, gender) and psychological factors (i.e., CORE-YP, DERS, RSES, MSPSS, INCOM, SMUint)] associated with latent class membership.

Data used for this study is available at an online repository hosted by the Open Science Framework at https://osf.io/dphny/?view_only=5bae7af428ff4f8a98143b9f12976968 with all the features needed to replicate this study.

## Results

### Trajectories of BSMAS, time spent on Instagram and time spent on TikTok

Three classes could be identified based on their joint scores on the Bergen Social Media Addiction Scale (BSMAS), time spent on Instagram and time spent on TikTok (hours). As shown in Table 2, the goodness-of-fit statistics suggest a four-class solution. Due to the remarkably small sample size of class 4 in the four-class solution, which is approximately 5%, the three-class solution was ultimately chosen on the basis of theoretical coherence and plausibility. It should be noted that for both models the last class has a relatively small sample size.

**Table 2:** Parallel Latent class analysis: Properties of the model tested for 2 to 4 classes.

	2-class model	3-class model	4-class model
**Fit statistics**			
** AIC**	16154.929	15945.190	15846.804
** BIC**	16278.896	16109.146	16050.750
** ssaBIC**	16180.530	15979.049	15888.922
**n (%) of participants**			
** Class 1**	278 (68.15%)	235 (58.31%)	241 (59.80%)
** Class 2**	125 (31.85%)	103 (25.56%)	105 (26.06%)
** Class 3**	–	65 (16.13%)	30 (7.45%)
** Class 4**	–	–	27 (6.70%)
** Entropy**	.781	.791	.840

**Fig 1:**
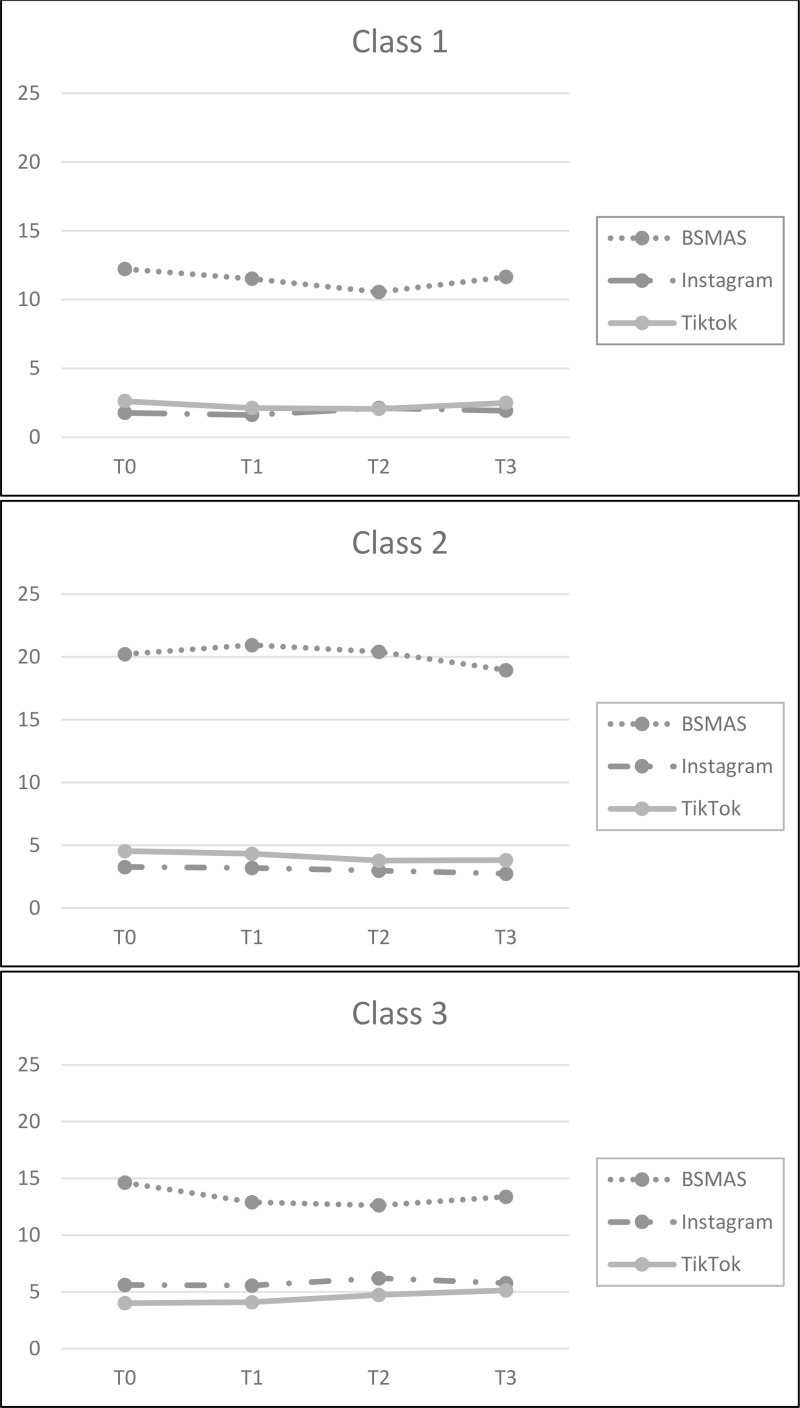
a – Class 1 – “Healthy user group” (58,31%), b – Class 2 – “Most vulnerable group” (25,56%) and c – Class 3 - “Engaged group” (16,13%). Note: BSMAS = Bergen Social Media Addiction Scale; Instagram = Time spent on Instagram (hours in a day); TikTok = Time spent on TikTok (hours in a day).

The baseline characteristics of the adolescents in all classes are presented in Table 3. Class 1 (n = 235, 58.31%), referred to as the “Healthy user group”, had the lowest levels of BSMAS (12.23 (0.30)), time spent on Instagram (hours) (1.78 (0.34)), as well as time spent on TikTok (hours) (2.62 (0.28)) out of the three classes. Baseline levels appeared to be unremarkable, showing a significant decrease in BSMAS scores from T0 to T2, but a subsequent significant increase at T3. Time spent using Instagram and TikTok (hours) tended to remain stable over time (see Table 4 and Fig 1.a).

**Table 3:** Baseline adolescents’ characteristics of all classes of the three-class-solution.

	Class 1 “Healthy user group”	Class 2 “Most vulnerable group”	Class 3 “Engaged group”
n (%)	235 (58.31%)	103 (25.56%)	65 (16.13%)
baseline characteristics			
** **sex [female], n (%)	104 (44.3)	66 (64.1)	39 (60.0)
** **age [years], M (SD)	15.64 (1.26)	15.71 (1.20)	16.09 (1.04)
** **BSMAS, M (SD)	12.23 (0.30)	20.21 (0.70)	14.62 (0.67)
** **Instagram time of use (hours), M (SD)	1.78 (0.34)	3.27 (0.47)	5.62 (1.32)
** **TikTok time of use (hours), M (SD)	2.62 (0.28)	4.54 (0.50)	3.99 (0.90)

Note: Instagram and TikTok time of use are described as hours in a day; BSMAS = Bergen Social Media Addiction Scale.

**Table 4:** Intercepts, liner slopes, quadratic terms (and standard errors) of the Parallel Latent Class Trajectory Groups for BSMAS, time spent on Instagram and time spent on TikTok (hours).

Class	Parameter	BSMAS	Time on Instagram (hours)	Time on TikTok (hours)
**Class 1**	Intercept	12.231 (.305)***	1.776 (.341)***	2.624 (.283)***
	Linear	−1.432 (.400)***	.039 (.331)	−.700 (.398)
	Quadratic	.402 (.136)***	.010 (.084)	.213 (.109)
**Class 2**	Intercept	20.215 (.697)***	3.274 (.469)***	4.540 (.504)***
	Linear	1.227 (.986)	−.100 (.711)	−.498 (.780)
	Quadratic	−.579 (.322)	−.022 (.225)	.086 (.245)
**Class 3**	Intercept	14.619 (.674)***	5.618 (1.315)***	3.988 (.904)***
	Linear	−2.135 (.966)*	.143 (1.655)	.218 (1.573)
	Quadratic	.577 (.311)	−.031 (.399)	.062 (.384)

BSMAS = Bergen Social Media Addiction Scale; * *p <*.05; *** *p <*.001.

Class 2, which could be described as the “most vulnerable group” (n = 103, 25.56%), showed the highest levels of BSMAS (M = 20.21 (0.70)), as well as time spent on TikTok (hours) (4.54 (0.50)) out of the three classes. The baseline levels of time spent on Instagram (hours) appeared to be higher (3.27 (0.47)) than in “healthy user group”. As shown in Table 4, all scores on the three scales tested tend to remain stable over time (see also Fig 1.b).

Class 3 (n = 65, 16.13%), which could be defined as the “engaged group”, consists of adolescents with average levels of BSMAS (14.62 (0.67)) and of time spent on TikTok (hours) (3.99 (0.90)) compared to the other two classes, but the highest baseline levels of time spent on Instagram (hours) (5.62 (1.32)) out of the three classes. Like time spent on TikTok (hours), time spent on Instagram (hours) appeared to remain constant over time. There was a significant decrease in BSMAS scores from T0 to T2 (Table 4 and Fig 1.c).

### Predictors of class membership

Multinomial logistic regression was then used to build a model of the relationship between baseline variables and membership of the three classes to identify potential predictors (i.e., “healthy user group”, “most vulnerable group” and “engaged group”).

**Fig 2:**
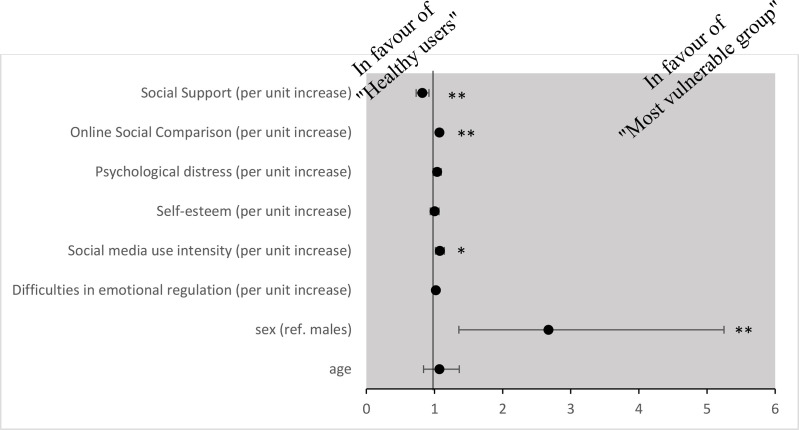
Results of multinominal logistic regression for “most vulnerable group” compared with “healthy user group”. Variables significantly associated with trajectories of BSMAS, time spent on Instagram and time spent on TikTok (hours in a day). Note: * *p < *.05; ** *p < *.01.

**Fig 3:**
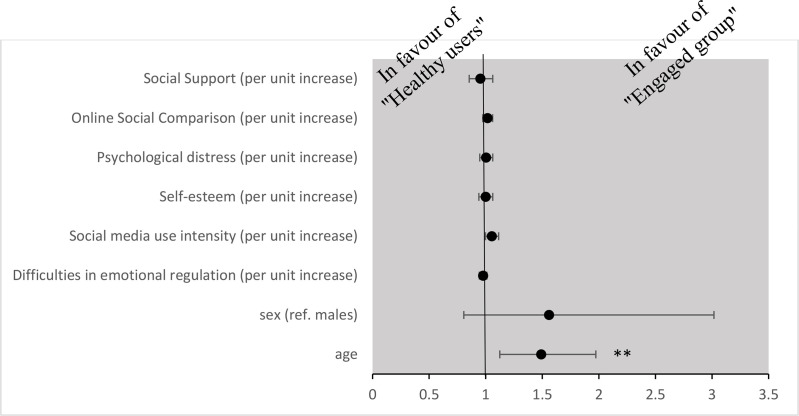
Results of multinominal logistic regression for “engaged group” compared with “healthy user group”. Variables significantly associated with trajectories of BSMAS, time spent on Instagram and time spent on TikTok (hours in a day). Note: ** *p < *.01.

Using multinomial logistic regression with class one (i.e., the “healthy user group”) as the reference group, it was found that female adolescents and those with higher INCOM and SMU intensity scores and lower social support were more likely to be in the “most vulnerable group” (Fig 2). It could also be shown that older adolescents have higher odds of belonging to the “engaged group” (Fig 3).

**Fig 4:**
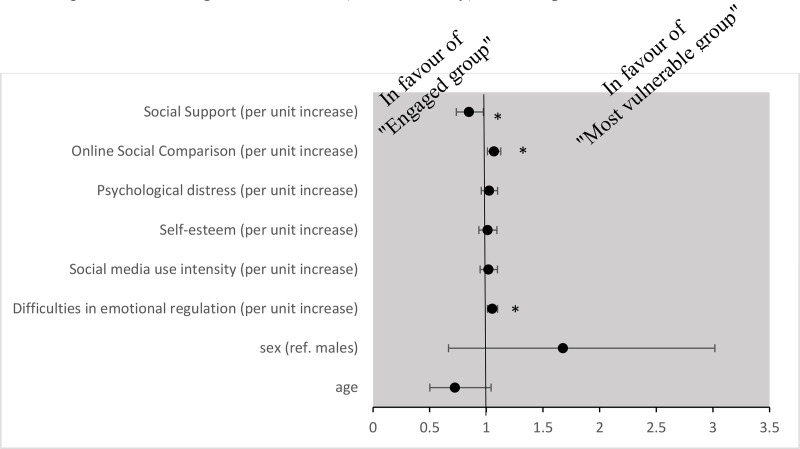
Results of multinominal logistic regression for “engaged group” compared with “most vulnerable group”. Variables significantly associated with trajectories of BSMAS, time spent on Instagram and time spent on TikTok (hours in a day). Note: * *p < *.05.

When comparing the “engaged group” and the “most vulnerable group” using class 3 “engaged” as the reference group to identify potential predictors of increased vulnerability, the multinomial logistic regression data showed that those with higher scores on DERS and INCOM, as well as those with less social support, had higher odds of being in the “most vulnerable group” (Fig 4).

## Discussion

The present study assessed the joint trajectories (co-development) of social media addiction and time spent on Instagram and TikTok in a group of Italian adolescents and identified a three-class model. The majority of the sample (58.31% of participants; ‘healthy user group’) showed a non-problematic pattern of social media use (with lower scores on the BSMAS out of the three classes and lower time spent on Instagram and TikTok). However, around 25.56% of participants (“Most vulnerable group”) reported a more risky profile, with both the highest level of social media addiction and time spent on TikTok (i.e., around 5 hours/day) out of the three classes, as well as moderate time spent on Instagram (i.e., around 3 hours/day). Adolescents with higher levels of social comparison and social media use intensity, as well as those with lower levels of social support, were more likely to belong to this profile of impaired functioning. These findings suggest that the role of social interactions (both online and offline) and social comparison tendencies as markers of problematic social media use should be further explored [36,59]. The role of weaker social support as a predictor of belonging to a high-risk versus low-risk class for problematic social media use confirms previous cross-sectional findings [60]. This finding should also be consistent with theoretical models [36,37] that suggest that shy and lonely individuals with low social support and poor interpersonal relationships may turn to social media to compensate for their lack of social skills. Such a mechanism may be particularly relevant during adolescence, a period characterised by complex developmental changes in both the process of identity formation and the development of social skills. Furthermore, women are more likely to belong to this profile of impaired functioning. Previous studies have investigated gender differences in social media addiction [61], with females spending more time on social media than males and being driven by different motivations (i.e., mainly for information and interpersonal communication) [62]. Moreover, a recent meta-analytic study confirms these gender differences across many cultures and countries [63].

Interestingly, adolescents in the third class ‘engaged group’ (16.13% of participants) reported moderate levels of problematic social media use, with high levels of Instagram use (i.e., around 6 hours/day) and levels of TikTok use quite similar to the ‘more vulnerable’ group (i.e., around 4 hours/day). This pattern was associated with older age. Considering the characteristics of the ‘most vulnerable’ and ‘engaged’ groups together, connection time does not seem to be sufficient to explain problematic social media use, a finding that is in line with previous studies [64]. More specifically, recent findings have shown that so-called screen time (‘how much’) is not enough to define one’s social media use as problematic, but that ‘how’ social media is used should also be considered. From this point of view, a high intensity of screen time and social media use may be normative in the daily lives of adolescents without negative effects on their mental health [18]. Our findings are consistent with those suggesting that problematic social media use is a heterogeneous problem [65], particularly among adolescents, and that different profiles of users can be detected [66]. Moreover, the current findings suggest that the high or low risk of problematic social media use cannot be based solely on the unique BSMAS scores. In this study, participants belonging to the classes 2 and 3 (41.69%) fall within the range of 14–26 scores, which is considered a moderate risk level for problematic social media use [67], but they report different patterns of social media use and different psychosocial characteristics. Further research is necessary to identify valid and reliable markers of problematic social media use among adolescents.

The current study identified some joint longitudinal trajectories of social media addiction and time spent on Instagram and TikTok among adolescents. In line with prior research [32], our findings suggest that problematic social media use showed persistently high or low trajectories. However, although the trends for time spent on social media (i.e., Instagram and TikTok) remained stable over time, the trend for BSMAS remained high and stable only for users in the ‘most vulnerable group’, confirming the moderate risk status of this group. Our finding regarding the differences in BSMAS scores over time in Class 2 and Class 3 suggests that problematic social media use may change over a short period of time for some adolescents, and is consistent with previous studies using the EMA methodology that showed a fluctuation in problematic internet use over the course of a few weeks [68]. We might speculate that some predisposing factors for social media addiction are at play. For example, it has recently been shown that peer rejection and peer popularity in social media can make adolescents more or less susceptible to social media addiction over a two-week period [69]. Thus, the role of online peer context should be further explored to identify predisposing factors for fluctuation in social media addiction [70]. Further research is also needed to examine whether the co-development of problematic social media use and time spent on social media differs over time, and whether the trajectories of social media use may parallel those of social media addiction. To date, studies on the development of problematic social media or internet use among the adolescents over the years have produced mixed results [31,32], and there is a call to distinguish between social media addiction and social media intensity, and to disentangle within-person and between-person effects to explain the heterogeneity of effects [71].

## Strengths and limitations

The study has a number of strengths, including differentiating the time of use of the two main social media (i.e., Instagram and TikTok) used primarily by adolescents. This is in line with the multidimensional model of social media use [22], which states that an overall or generic measure of social media use is not sufficient, but information should be collected on social media activities, motives that lead people to use social media, and main communication partners. All these characteristics are different for different social media. In addition, the longitudinal design of the study allows us to go beyond the results of previous studies that have used a cross-sectional design to classify different profiles of problematic social media use, thus allowing us to examine the stability of these profiles over time. However, there are several limitations. First, the small sample size. In this study, the four-class solution was excluded due to the small sample size of one class (close to the commonly accepted threshold of 5%). The joint trajectories of this study should be replicated with a large sample size. Second, the short interval between waves (from one to four months). Therefore, our results should be replicated with longer intervals between waves in order to verify their stability over time and at different stages of adolescents’ lives. Another limitation is related to the use of self-report measures, that are subject to recall and social desirability biases. Moreover, we used the Bergen Social Media Addiction Scale to measure participants’ level of addictive use of social media. However, this measure has been criticized by some authors (e.g., [72]) for its questionable theoretical and methodological basis and its validity in distinguishing between engaged and pathological behavior. Thus, future research should adopt a broader focus in assessing problematic social media use, using measures that are not simply embedded in an addiction model. In terms of data collection, although in the current study we asked participants to report objective tracking data from IOS and Android devices, self-reporting bias cannot be excluded. In future studies, social media data could be collected through custom applications or application programming interfaces to access data on user behavior and limit reporting bias [26]. Also, although this study was not conducted during the restrictions imposed by the COVID-19 outbreak, some empirical studies have shown that the COVID-19 pandemic has further increased the intensity and problems of social media use (e.g., [73]). We cannot exclude the possibility that some of the patterns of use recorded in this study may still represent long-term effects of this pandemic-related increase in problematic social media use. Finally, due to the lack of an experimental design, we cannot rule out the possibility that other variables may have influenced the fluctuations of the BSMAS between time points.

Despite these limitations, our study provides preliminary support for identifying joint trajectories of problematic social media use and time spent on social media among adolescents and suggests that individual differences should be explored for their contribution to a more or less “healthy” use of online social media platforms among adolescents.
